# Electrolytic Cleaning and Regenerative Therapy of Peri-implantitis in the Esthetic Area: A Case Report

**DOI:** 10.1055/s-0042-1750773

**Published:** 2022-07-04

**Authors:** Francesco Gianfreda, Andrea Punzo, Valeria Pistilli, Patrizio Bollero, Gabriele Cervino, Cesare D'Amico, Francesco Cairo, Marco Cicciù

**Affiliations:** 1 Oral Pathology Department, University of Rome Tor Vergata, Italy; 2 Department of Biomedical and Dental Sciences, Morphological and Functional Images, School of Dentistry, University of Messina, Policlinico G. Martino, Via Consolare Valeria, Messina Italy; 3 Department of Biomedical and Dental Sciences, Morphological and Functional Images, University of Messina, G. Martino Polyclinic, Messina, Italy; 4 Department of Experimental and Clinical Medicine, Research Unit in Periodontology and Periodontal Medicine, University of Florence, Florence, Italy

**Keywords:** guided tissue regeneration, periodontics, oral surgery, peri-implantitis

## Abstract

Implantology represents the gold standard in oral rehabilitation. Unfortunately, a new pathology begins to show itself to clinicians that no longer affects only and solely the supporting tissues of the tooth but also dental implants and peri-implantitis. In this study, we present a case report regarding a tissue regeneration maneuver involving dental implants. The clinical and radiographic results are encouraging, regarding the use of these techniques on implant surfaces. Surely, the advent of new biomaterials and surgical techniques will make this practice safe and predictable.

## Introduction

The insertion of dental implants in clinical practice necessarily involves facing, statistically, a variable number of cases of mucositis and peri-implantitis.

Mucositis is a reversible inflammatory process, limited to the peri-implant soft tissue, without affecting the attachment epithelium or the bone tissue. 1

Peri-implantitis, on the other hand, is an inflammatory process which, in addition to affecting the soft tissues, is associated with peri-implant bone loss. 2 Both pathological manifestations have a bacterial etiology, that is, they are caused by biofilms that colonize the surfaces of the implant fixture and the abutment. 3 4

Several studies have evaluated the incidence of peri-implantitis. According to Derks et al, 5 45% of patients with implants are affected by peri-implantitis. In contrast, Atieh et al 6 and Rakic et al 7 obtained results in agreement with percentages of 18.8 and 18.5%, respectively. On the percentage differences reported by these studies, it is necessary to clarify that the authors evaluated different threshold values in the definition of pathology. To standardize the concept of peri-implantitis, in the 2017 World Workshop on the Classification of Periodontal and Peri-Implant Diseases and Conditions, a higher probing than baseline associated with clinical signs of inflammation was reported as a pathological clinical sign. 8 Peri-implantitis is an inflammatory process which affects the tissues around the osseointegrated implant leading to a loss of the supporting bone tissue. The cause is either infectious or it is an incorrect implant-prosthetic passivation (occlusal trauma), although both causes can be found clinically. The radiographic examination allows to easily distinguish an occlusal trauma peri-implantitis from a bacterial one.

To safeguard the osseointegration process, therapies are used that include the use of antibiotic and anti-inflammatory drugs, as well as a possible revision of surgical curettage of the affected area, and also guided bone regeneration (GBR). All therapies aim to eliminate bacteria from the surface of the implant both in the covered (without affecting the gum) and in the open (incising the gum and lifting it). When inflammation is present, therefore, it is considered necessary to use methods to decontaminate implant surfaces, with the goal of prolonging the prognosis of rehabilitations. In fact, the complete replacement of affected implants is not indicated, both in terms of morbidity and costs. Instead, we should aim at the development of predictable methods to restore implant health.

Several methods of implant decontamination have been suggested, including mechanical therapies aimed at removing bacterial biofilm and smoothing the involved implant surfaces and chemical therapies such as application of antiseptics and local and systemic antibiotics. However, there is no consensus on the most effective protocol for detoxification of implant surfaces.

Mechanical removal of bacterial aggregates on the surfaces, using powder spray with erythritol particles, accelerated by air pressure, is a method that is positively reported in the literature with regard to clinical improvements in depth and bleeding on probing. 9

It has also been demonstrated that local antimicrobial agents play an important role in peri-implant bacterial control, particularly doxycycline, a bacteriostatic molecule belonging to the tetracycline class which is inserted into the peri-implant pocket and can be effective for up to 14 days. 10

Another critical site that easily colonized by bacteria and difficult to reach for decontamination is the fixture-abutment interface, in this regard do Nascimento et al 11 demonstrated how the application of iodoform paste in the fixture-abutment connection has a decisive role in preventing bacterial colonization.

However, the exclusively nonsurgical approach was inadequate for the treatment of moderate-to-severe peri-implantitis where a surgical approach is instead required to reach the deepest implant surfaces to be decontaminated to restore the anatomy, to eliminate the peri-implant pockets, and to perform a regeneration of the present bone defect.

It has been shown that vertical peri-implant bone defects can and should be treated by regenerative surgery, using only autologous or heterologous bone in combination or not with a membrane (GBR) and growth factors. 12

Studies have evaluated that platelet concentrates, obtained by centrifugal separation of venous blood which include growth factors, are involved in increased angiogenesis activity, stimulate fibroblast and osteoblast activity, and increase hard and soft tissue regeneration. 13 A study conducted by Schlee et al investigated the efficacy of using electrolytic decontamination using GalvoSurge (GS1000, GalvoSurge Dental AG, Widnau, Switzerland) in association with spray treatment of implant surfaces and without spray treatment of implant surfaces. 14

The aim of this study is to present the management of a dental implant clinical case in the esthetic zone (left maxillary central incisor; 2.1) affected by peri-implantitis, with a combination of both chemical and surgical techniques to restore implant health.

## Case Report

This case report describes a clinical case of a patient, 62-year-old male, Caucasian, in good general health, without any systemic problems, nonsmoker, and not on medication (ASA1: a normal healthy patient. Example: Fit, nonobese [BMI under 30], a nonsmoking patient with good exercise tolerance).

The patient has an implant (Bredent, Senden, Germany) in zone 2.1 placed with a postextraction technique with immediate loading in 2014.

**Fig. 1:**
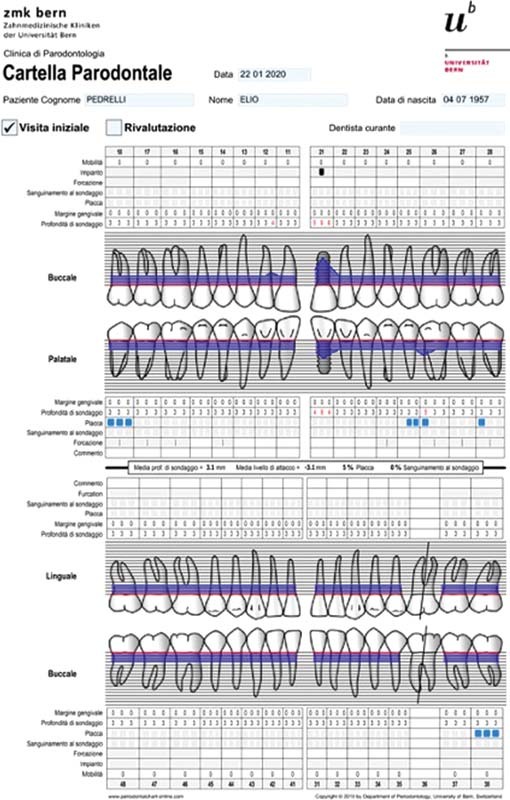
Periodontal chart at baseline.

The patient reported that he underwent 6-month maintenance therapy and, in fact, from the compilation of the periodontal chart and radiographic evaluation (full endoral), a good periodontal health is described with only one >5-mm probing in the mesiopalatal site of tooth element (left maxillary first molar 2.6), corresponding to a prosthetic restoration and with slight plaque accumulation. A good oral hygiene status is noted given the absence of bleeding at the probing and a plaque index of 5%. In correspondence of the implant in zone 2.1, on the other hand, pathological probes were found, especially in the central vestibular site with PD = 9 mm ( Fig. 1 ).

**Fig. 2:**
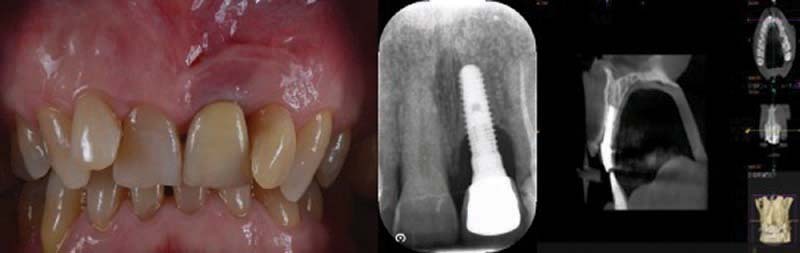
Clinical and radiographic image (2D, 3D) at baseline. 2D, two-dimensional; 3D, three-dimensional.

Periapical X-ray showed peri-implant bone resorption and cone-beam computed tomography (CBCT) showed a 5.5-mm circumferential deficit ( Fig. 2 ).

Clinically, the texture of the peri-implant mucosa is edematous, violet colored, but without loss of substance and/or exposure of the implant coils. Occlusal vision shows a clear deficit of mucogingival volume and/or bone in the vestibular side of the 2.1 area ( Fig. 2 ).

We proceed with the treatment starting with a professional oral hygiene session, with decontamination of the implant area with erythritol Aereosol through a dedicated disposable tip inserted in the peri-implant defect itself (AIR-FLOW Master Piezon; EMS, Nyon, Switzerland).

**Fig. 3:**
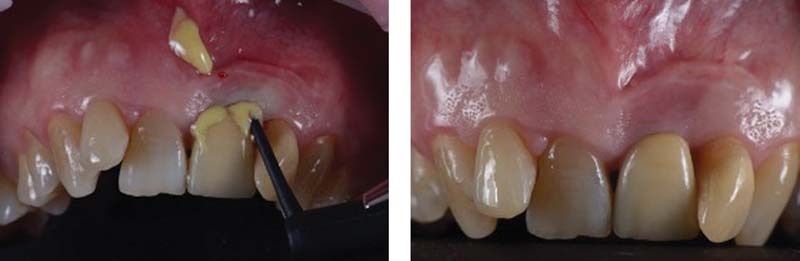
( **A, B** ) application of antibiotic gel.

Subsequently, PERIOSTAT gel (iclated doxycycline) is applied, via a carrier with a tip, within the defect 10 ( Fig. 3 ).

**Fig. 4:**
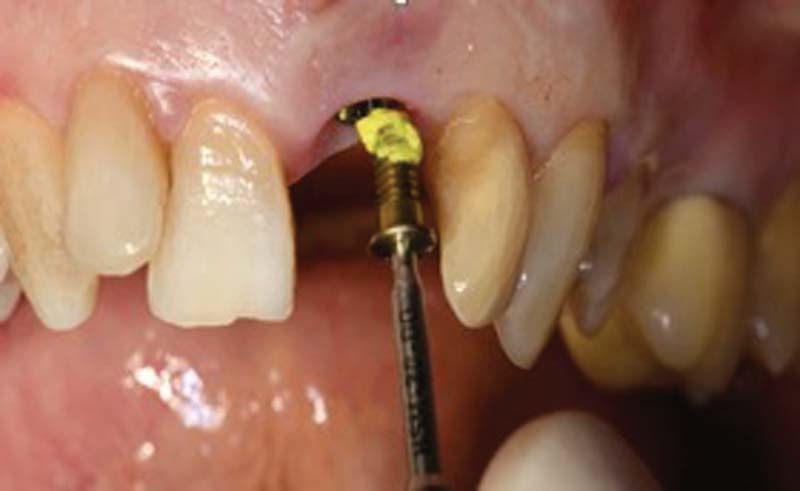
Insertion of the cap screw with iodoformic paste.

After 1 week from decontamination, the crown on implant 2.1 is removed, the cap screw is inserted with application of iodoform paste 11 and then a maryland bridge has been placed ( Fig. 4 ).

**Fig. 5:**
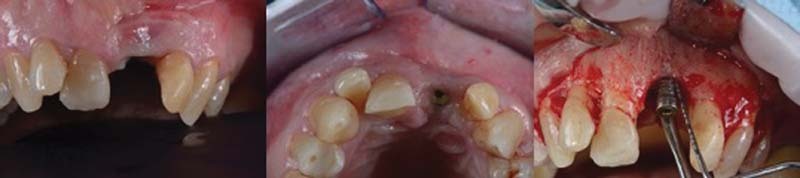
Surgical exposure of the peri-implant site.

Once the soft tissues have healed, after 4 weeks, we can proceed with a regenerative surgery to regenerate the portion of peri-implant bone tissue lost due to peri-implantitis ( Fig. 5 ).

Once anesthesia with vasoconstrictor (1:100,000) is performed, a flap is incised according to Canullo et al. 12

Vestibularly, the design of the flap is similar to a flap for the treatment of multiple recessions in mucogingival surgery therefore it involves the following:

- A crestal incision.
- Incisions to draw surgical papillae bilaterally up to three teeth distal from the area to be regenerated.
- Flap dissection at half thickness up to the amelocemental junction and at a full thickness apically to it.
- Deepithelialization of the anatomic papillae to create a connective receiving bed for the surgical papillae.
- The lateral extension of the incisions allows to have a passivation of the flap without the need of vertical unloading cuts.

**Fig. 6:**
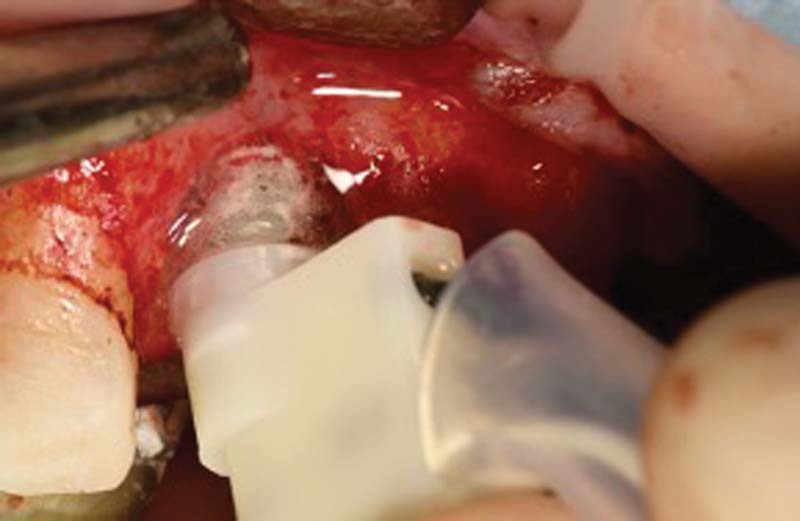
Decontamination using GS1000 GalvoSurge Dental AG.

Once the flap has been removed, the defect degranulated and washed with rifampicin (RIFADIN, Sanofi, Milan, Italy), the implant is decontaminated using an electrolytic approach using GS1000 GalvoSurge Dental AG 14 ( Fig. 6 ).

**Fig. 7:**
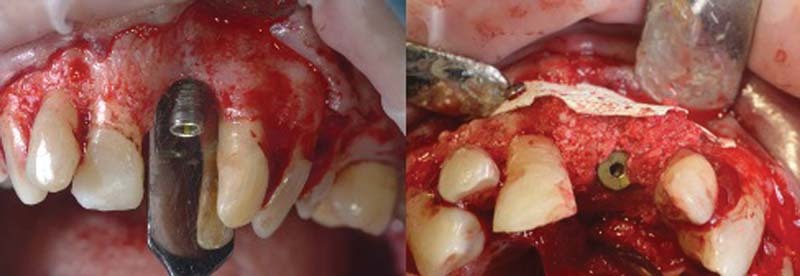
Defect filling.

Once the electrolytic decontamination is completed, the bone defect is filled with a deproteinized bone graft (Bioss, Geistlich Pharma AG, Wolhusen, Switzerland) mixed with autologous bone harvested intraorally and then covered with a resorbable membrane (Cytoplast Ti-250, Deore Materials, Osteohealth, New York, United States; Fig. 7 ).

**Fig. 8:**
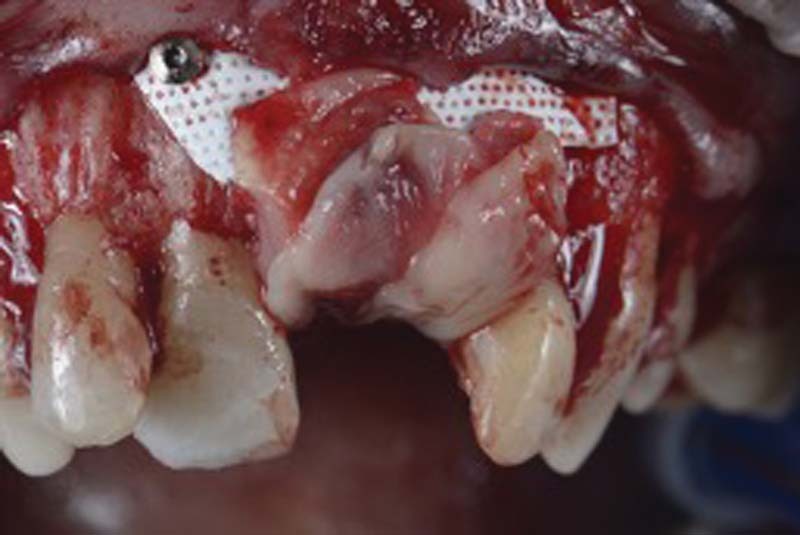
Membrane fixation with two mini screws and insertion of platelet aggregates.

The membrane is then fixed with two metal mini-screws and platelet aggregates are inserted to promote healing 13 ( Fig. 8 ).

Once the regenerative phase is completed, the flap is further passivated through periosteal incisions apical to the flap and then sutured to the anatomic papillae.

**Fig. 9:**
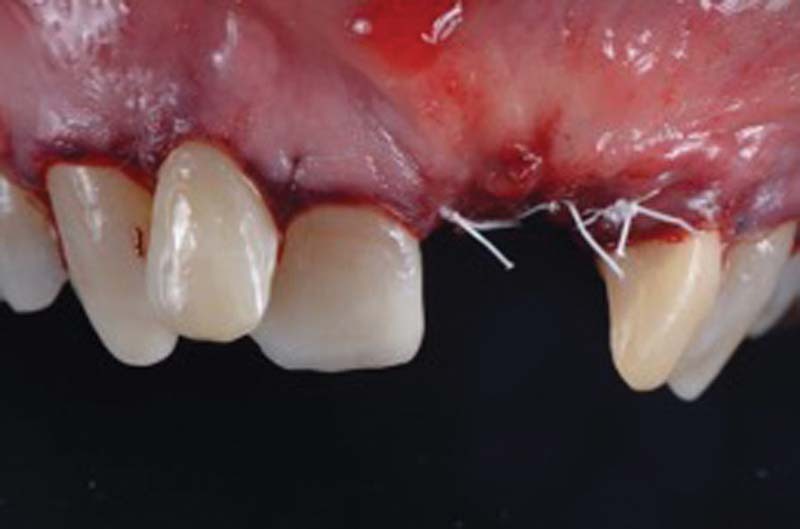
Sutures.

Sutures: PTFE 5.0 (Omnia, Fidenza, Italy; mattress suture on the implant area) and PGCL 6.0 (Monofast, Kirkinil, Greece; detached stitches and sling stitches on the papillae; Fig. 9 ).

The patient is instructed in postsurgical oral hygiene maneuvers as follows:

- Avoid brushing the area of surgery until suture removal (14 days).
- To rinse after the first 24 hours with CHX mouthwash (Curasept, Saronno, Italy) at 0.12% during the same period of time twice a day.

Prescribed: amoxicillin of 875 mg + 125 mg Ac. Clavulanic for 4 days 2 times a day and nonsteroidal anti-inflammatory drugs (NSAIDs) as needed.

**Fig.10:**
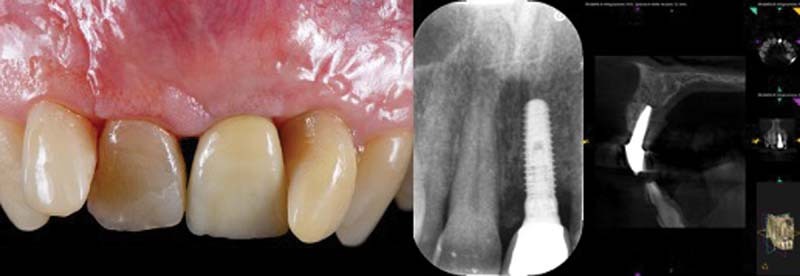
Clinical and radiographic (2D, 3D) follow up at 1 year. 2D, two-dimensional; 3D, three-dimensional.

The patient is checked every 7 days during the first month and then monthly for 12 months, when a new CBCT is then performed ( Fig. 10 ).

## Results

During the numerous controls performed in the first 12 months after surgery, no clinical problems were detected, with an excellent control by the patient of home hygiene and a stability of periodontal indices, completely within the physiological range.

There is a gingival recession, especially on the distal side in relation to the lack of bone peak on the lateral incisor. At 12 months, both an endoral periapical control X-ray and a CBCT were performed, showing a complete circumferential filling of the treated peri-implant defect. In fact, a total bone coverage of the shoulder of the 2.1 implant can be seen, with a bone gain of 6 mm on the mesial aspect and 5 mm on the distal aspect.

## Discussion

The treatment of peri-implant lesions has long been a controversial topic. The purpose of the present case-report was to define a new surgical and regenerative protocol to restore peri-implant tissue health and esthetics in the esthetic zone. The results of the follow-up of more than 2 years showed an excellent reosteointegration of the bone around the implant affected by a combined defect similar to a crater in the most apical part and a vertical defect in the distal region.

It is important to emphasize that the result obtained from the clinical case is the sum of a series of biological and mechanical factors that a clinician should consider. First of all, a nonsurgical and antibiotic approach was used to treat the periodontal pocket that presented bleeding and suppuration phenomena. The aim of this first approach is also to make the tissues less edematous and more manageable from a surgical point of view. 10

Microbiological studies conducted by Canullo et al 3 have shown that the implant connection, being an oxygen-poor site, tends to select anaerobic gram-negative bacteria. This, during the life of the implant, especially in less stable connections or incongruous prosthetic restorations, leads to a micropumping phenomenon that results in an increased risk of peri-implantitis. For this reason, 4 weeks before performing the regenerative surgical therapy, it was necessary to perform a removal of the previous prosthetic artifact and at the same time, iodoform paste was inserted together with a new cap screw. 11

It is important to underline that the removal of the crown allows from a clinical point of view a better access to the pocket and to the surface to be decontaminated.

When approaching a vertical regenerative case, one of the most complex aspects is certainly the management of the flap. For this reason, in this case, a platelet concentrate has been wrapped around the screw cap with the aim to enhance the healing of soft tissues. In addition, some studies in the literature have drawn attention to the fact that the presence of leukocytes in platelet concentrates may allow a greater control of postoperative infections and may be a protective factor in cases at risk of osteomyelitis. 15 16

The surgical therapy used is initially characterized by the use of a coronally advanced flap for the treatment of multiple recessions. This allows for the avoidance of scarring in areas where release cuts might be necessary and ensures a greater blood supply to the flap.

Total decontamination of the surfaces is required before regenerative surgery is performed. The presence of nano- and microparticles, bacterial-derived antigens, or ions on implant surfaces can lead to bone resorption or failure of osseointegration. To fully understand these biochemical mechanisms, osteoimmunology, a rapidly developing branch that studies the interrelationship between bone tissue cells and the immune system, has been introduced. 17

Indeed, initially the inflammatory infiltrate together with macrophages allows for a proregenerative peri-implant environment. Subsequently, if the inflammation is not resolved or reactivated due to the immunogenic signals of the contaminants, infections with subsequent bone loss or aseptic inflammation could occur. 17

Nonsurgical methods of surface decontamination have demonstrated suboptimal results. 18

Methods of implant surface decontamination using mechanical, sonic, and ultrasonic scalers, laser, air flow, and various chemical solutions, such as chlorhexidine digluconate, citric acid, and hydrogen peroxide, have been proposed in many studies. 19 20 21 In addition, interesting results have been obtained with the use of plasma argon. 22

However, in the literature, results about newly formed bone vary widely, and it is often difficult to quantify how much of the radiographically present bone is actually osseointegrated on the implant surfaces.

Small areas of osteitis around the body of implants in peri-implantitis may remain contaminated despite mechanical removal of granulation tissue, so it may be useful to treat contaminated bone by topical application of rifampicin.

Once the granulation tissue around the implant was removed and the recipient bone site was exposed, a new tool called GalvoSurge was introduced to decontaminate the surface. The recently developed system involves using an electric current on an implant treated with sodium formicate. Hydrogen anion (OH − ) and cation (H ^+^ ) are dissociated by the current. The highly reactive H ^+^ ions form hydrogen bubbles on interaction with the stray electrons which lift the biofilm from the implant surface. 13 An *in vitro* study demonstrated complete sterilization of surfaces after electrolytic treatment. 23

An extremely interesting aspect is represented by the possibility to decontaminate an implant surface without changing its surface microtopography and therefore without affecting its physical properties.

Promising reosteointegration of contaminated implant surfaces after electrolytic cleaning has been demonstrated in two clinical studies. 17 24 25 26 27

## Limitations

The limitations of this technique are represented by the impossibility to regenerate defects above the bone peaks, so a combined regenerative and resective approach should not be underestimated in cases that require it. The use of GalvoSurge in fact would allow the removal of particular matter resulting from the release of titanium particles around the surface without affecting the healing process. In the literature, there is much debate about the influence of metal particles on tissues. In fact, this same particulate could result in a negative modulation of osteoimmunology and could result in an aseptic foreign body reaction around the implant.

Ultimately, it can be said that electrolytic cleaning, although recently introduced, is an extremely interesting method for the regenerative management of peri-implants. Future studies will be needed to understand the dynamics around healing of sites treated with GalvoSurge.

Nonmechanical therapies involve the use of antiseptic and antibiotic solutions, both in the form of washes and in slow-release forms from the gel to the impregnated fibers. The laser can sterilize surfaces. The common limitation of all therapies that are limited to eliminating bacteria by disinfecting or sterilizing is that they will recolonize the spaces as bacteria are not found only on implants. It becomes essential to correct these bone defects, so that they do not return to being reservoirs of infection. GBR is useful in this sense, unless you resort to resective therapies that will bring decontaminated surface outdoors into cleanable areas. It must be observed, however, how important it is to intercept, together with the microbiological contamination, the possible iatrogenic background of the peri-implant disease. Failing in classifying the possible surgical or prosthetic triggering factors may lead to a failure in the treatment outcomes. 28 29 30 31 32 33 34 35 36 37 38 39 40 41 42

## Conclusion

The presented case demonstrated a new and promising protocol for the regenerative management of sites affected by peri-implantitis. The ability to regenerate healthy, viable bone around implants is critical in cases where esthetic demands are more demanding. Combining these surgical techniques and these pharmacological protocols will allow clinicians to offer safer and more predictable rehabilitations over time. Future studies will be necessary to fully understand the timing and dynamics regarding wound healing of sites treated with GalvoSurge. In addition, a comparison of the healing dynamics of treated sites in relation to different implant surfaces would be desirable.
